# Violation of rhythmic expectancies can elicit late frontal gamma activity nested in theta oscillations

**DOI:** 10.1111/psyp.13909

**Published:** 2021-07-26

**Authors:** M. Edalati, M. Mahmoudzadeh, J. Safaie, F. Wallois, S. Moghimi

**Affiliations:** ^1^ Inserm UMR1105 Groupe de Recherches sur l’Analyse Multimodale de la Fonction Cérébrale CURS Amiens France; ^2^ Electrical Engineering Department Ferdowsi University of Mashhad Mashhad Iran; ^3^ Inserm UMR1105 EFSN Pédiatriques CHU Amiens sud Amiens France

**Keywords:** event‐related potential, mismatch negativity, phase‐amplitude coupling, prediction error, predictive coding

## Abstract

Rhythm processing involves building expectations according to the hierarchical temporal structure of auditory events. Although rhythm processing has been addressed in the context of predictive coding, the properties of the oscillatory response in different cortical areas are still not clear. We explored the oscillatory properties of the neural response to rhythmic incongruence and the cross‐frequency coupling between multiple frequencies to further investigate the mechanisms underlying rhythm perception. We designed an experiment to investigate the neural response to rhythmic deviations in which the tone either arrived earlier than expected or the tone in the same metrical position was omitted. These two manipulations modulate the rhythmic structure differently, with the former creating a larger violation of the general structure of the musical stimulus than the latter. Both deviations resulted in an MMN response, whereas only the rhythmic deviant resulted in a subsequent P3a. Rhythmic deviants due to the early occurrence of a tone, but not omission deviants, seemed to elicit a late high gamma response (60–80 Hz) at the end of the P3a over the left frontal region, which, interestingly, correlated with the P3a amplitude over the same region and was also nested in theta oscillations. The timing of the elicited high‐frequency gamma oscillations related to rhythmic deviation suggests that it might be related to the update of the predictive neural model, corresponding to the temporal structure of the events in higher‐level cortical areas.

## Abstract 2

Rhythm is an important feature of both music and speech. Rhythm perception involves complex cognitive processing, including shaping expectations about the upcoming events in the auditory stream. In the context of predictive coding, we show that while processing musical rhythmic incongruences, the cross frequency interaction between slow and fast oscillators and coordinated short‐term gamma oscillations over the frontal area are involved in the update of the neural model of the rhythmic structure.

## 1 INTRODUCTION

Music consists of organized sequences of sounds, arranged in hierarchical temporal patterns that unfold over time and involve complex cognitive processes (Patel & Daniele, 2003; Pearce & Wiggins, 2012). Music perception involves the generation of expectations, anticipation of their development, and eventually violation or fulfilment of the predictions (Cheung et al., 2019; Friston, 2002, 2010; Gold et al., 2019; Lumaca et al., 2019). Generally speaking, music plays with our expectations in two forms; it plays with *what* to expect and *when* to expect an event. Although the part of *what* to expect is shaped through melody, phrase, and harmonic structures, the part of *when* to expect an event involves matching the rhythmic structure of music with rhythmic or metrical templates that can be extrapolated in the future (Rohrmeier & Koelsch, 2012).

Recently, music perception has been addressed in the context of predictive coding (Bouwer et al., 2020; Cheung et al., 2019; Friston, 2002, 2005; Gold et al., 2019; Koelsch et al., 2019; Rohrmeier et al., 2012), which provides a compelling framework to address the predictive processes. This view is based on hierarchical Bayesian inference (Friston, 2005, 2010) and assumes that the brain constantly models statistical regularities in the auditory stream, actively produces predictions that are compared with incoming auditory inputs, and optimizes representations to reduce prediction error (Koelsch et al., 2019). It has been suggested that when exposed to music, the listener's brain extracts the temporal regularities, as well as the rhythmic structures, and shapes a probabilistic model, which in turn provides predictions on *when* to expect an event (Koelsch et al., 2019; Lumaca et al., 2019; Vuust et al., 2009). If the auditory input violates the prediction of the neural model, e.g. if a certain note arrives earlier/later than expected, an *error* signal is generated—the more accurate the prediction, the smaller the prediction error (Hansen & Pearce, 2014). The brain is functionally organized to minimize this *error* to accomplish temporally precise predictions. This attempt is shaped by a reciprocal cascade of cortical functions, in which higher‐level structures generate predictions of inputs from lower‐level ones and pass them through top‐down connections. Then, error signals are transferred through bottom‐up connections to update the models that led to these predictions (Kanai et al., 2015).

The neural response to violations of rhythmic structures has been addressed mostly in the context of specific auditory evoked potentials, the mismatch negativity (MMN) (Bouwer & Honing, 2015; Bouwer et al., 2014; Geiser et al., 2009; Grahn, 2012; Honing et al., 2009; Lappe et al., ,^2013^, ^2016^; Lelo‐de‐Larrea‐Mancera et al., 2017; Vuust et al., 2016; Zhao et al., 2017), as well as later ERP components, including P3 (Friedman et al., 2001). Rhythmic deviations, in terms of a tone occurring earlier than expected, elicit an MMN between 100–200 ms (Lumaca et al., 2019), which, depending on the experimental design, can be followed by a subsequent P3a in a time window of ~200–300 ms (Geiser et al., 2009; Vuust et al., 2009, 2016). In addition, the pre‐attentive neural response to the occasional omission of tones within a rhythmic sequence manifests as an MMN response (Bouwer et al., 2014; Honing et al., 2009; Ladinig et al., 2009), which is followed by a P3a component in some paradigm designs (Bouwer et al., 2016). The theory of predictive coding has been used to explain the neural response to rhythmic incongruence, with the MMN having the properties of an error term and the P3a reflecting upward propagation and subsequent evaluation of the error across higher levels of the hierarchy in the neural structures (Vuust et al., 2009). In this view, the relatively less pronounced MMN response to small rhythmic violations in complex rhythmic patterns has been related to less confident predictions of the weaker neural models—the more difficult the stimuli are to model, the weaker the predictive models, and hence the smaller the prediction error (Lumaca et al., 2019). Consistent with this view, it has also been demonstrated that the amplitude of the MMN depends on the metrical position of the omitted tone, being stronger for metrically stronger positions than metrically weaker ones (Bouwer et al., ,^2014^, ^2016^; Ladinig et al., 2009), again eliciting a more pronounced MMN in response to larger violations. Furthermore, the amplitude of the MMN is modulated by musical training (Lappe et al., 2011, 2013) and is stronger in musicians than non‐musicians (James et al., 2012; Vuust et al., 2009; Vuust et al., 2005), probably reflecting a stronger metrical predictive structure in experienced listeners. In addition, it has been suggested that musical cultural backgrounds shape expectations toward rhythmic structures implicitly through music exposure throughout life, which in turn can modulate the error signal created in response to rhythmic incongruence (Akrami & Moghimi, 2017; Haumann et al., 2018). Put together, the statistical regularities and hierarchical nature of rhythmic structures make music rhythm a powerful tool to investigate predictive coding in the brain, which in turn can be employed to explain the neural dynamics underlying the perception of rhythm.

The mechanisms underlying the reciprocal relationships between predictions and prediction errors have been investigated using several experimental paradigms that rely on the contrast between neural responses to anticipated and novel auditory stimuli (Garrido et al., 2007, 2008). An elegant paradigm, referred to as the ‘‘local‐global’’ paradigm, has been developed and used to address the auditory novelty response, as well as dissociate predictions based on local probabilities from those related to global rules (Chao et al., 2018; Chennu et al., 2013). It has been demonstrated that the detection of the violation of the global rules, in which subjects have to create a neural model of the temporal pattern and non‐local dependencies of tones, results in a more global and integrative violation of expectation, which manifests as both early and late ERP components (Chennu et al., 2016). Violations of the global rule of stimuli sequence also elicit widespread and protracted oscillatory responses (Dürschmid et al., 2016), including low‐frequency theta/alpha effects (Recasens et al., 2018), fronto‐temporo‐parietal depression in the beta‐band (Dürschmid et al., 2016), and high gamma augmentation in the temporal and frontal areas (Karoui et al., 2015; Kaiser et al., 2005, 2007). The different oscillatory activities over distinct brain regions, as well as early/late ERP components during the processing of low‐level violations versus violations of the global rules, reflect the different underlying neural mechanisms recruited for the aforementioned processes. In the context of this paradigm, the response to a deviant tone has also been compared to that of the omission of an expected tone (Wacongne et al., 2011). It has been demonstrated that the relatively late ERP component (200–300 ms) present in the processing of a deviant tone (in which the stimulus differs from that predicted by the recent history of the stimuli) is not elicited during the processing of omission (lack of any sensory input) (Chennu et al., 2016).

Generally, the predictive coding framework suggests that lower‐level violations arise from the primary auditory cortex, whereas violations of the global rule of sequences, involve (1) revising the mental representation of the sequence in the higher‐level system, including the prefrontal cortex (Bekinschtein et al., 2009; Chao et al., 2018; Chennu et al., 2013; El Karoui et al., 2015; Uhrig et al., 2014), and (2) updating the predictions for the next trial in lower‐level sensory areas (Chao et al., 2018). Deviant auditory stimuli evoke neural responses in the bilateral auditory cortex, superior temporal gyri, and prefrontal cortex (Doeller et al., 2003; Molholm et al., 2005; Rinne et al., 2005). Beyond the auditory cortex, the prefrontal cortices integrate error signals to update the prediction models (Bastos et al., 2012; Summerfield et al., 2006). Indeed, there is evidence for a frontotemporal hierarchy of prediction and prediction error information transfer (Chao et al., 2018; Chennu et al., 2016; Garrido et al., 2007, 2008, 2009; Phillips et al., 2015).

Music rhythmic violations are a good example of the violation of the global rule of sequences, in which the expectation is developed based on the modeled local and non‐local temporal dependencies. We designed an experiment to investigate the neural response to rhythmic deviations in which the tone either arrived earlier than expected or the tone in the same metrical position was omitted. These two manipulations modulate the rhythmic structure differently, with the former creating a larger violation of the general structure of the musical stimulus than the latter, in which the temporal Gestalt characteristics of the chord sequence (corresponding to the rhythmic or relative temporal pattern of acoustic events that leads to their perceptual grouping Koelsch, 2011; Tenney & Polansky, 1980) ) are not violated. Although the processing of music rhythm has been addressed in the context of predictive coding, the properties of the oscillatory response in different cortical areas are still not clear. To date, mostly ERP components have been analyzed to address the neural mechanisms of rhythm perception. To better understand the mechanisms underlying the neural response to rhythmic incongruence and the perception of rhythm, we explore the oscillatory properties of these responses and compare them under the two conditions of manipulation. In addition, growing evidence suggests that perception involves cross‐frequency coupling (CFC) in terms of coordinated slow and fast neural oscillations, typically nested theta/gamma oscillations (Buzsáki & Draguhn, 2004; Canolty et al., 2006; Lakatos et al., 2005), which presumably enhance combinatorial opportunities for encoding (Rasch & Born, 2013) and facilitate synaptic plasticity (Bergmann & Born, 2018; Buzsáki & Draguhn, 2004; Salimpour & Anderson, 2019). Thus, given the role of CFC in perception, we explore the CFC between multiple frequencies to explain the underlying mechanisms involved in rhythm perception. Our central hypothesis is that there should be a fundamental difference in the neural response to violations consisting of the omission of tones and rhythmic violations due to tones arriving earlier than expected, with the latter creating a larger violation of the rhythmic structure. We hypothesize that the greater violation of the rhythmic structure elicits stronger late oscillatory activities following the MMN (which in turn reflects the prediction error), which is related to the update of the neural model of the rhythmic structure.

## 2 METHOD

### 2.1 Participants

Fourteen healthy right‐handed volunteers (age 20 ± 2 years, 7 females) participated in the study after providing written informed consent. Participants were non‐musicians with a similar educational background (undergraduates or MSc students, with less than 3 years of musical activity), normal hearing, and normal or corrected‐to‐normal vision. They reported normal nocturnal sleep patterns (7–9 hr starting between 10 p.m. and 12 a.m.) for the week before the experiment. They had not used caffeine, nicotine, or energy drinks on the day of the experiment and had not performed excessive exercise within the previous 24 hr. As assessed by a questionnaire, participants had no history of neurological or psychiatric disorders.

### 2.2 Auditory stimuli and the experimental paradigm

**FIGURE 1:**
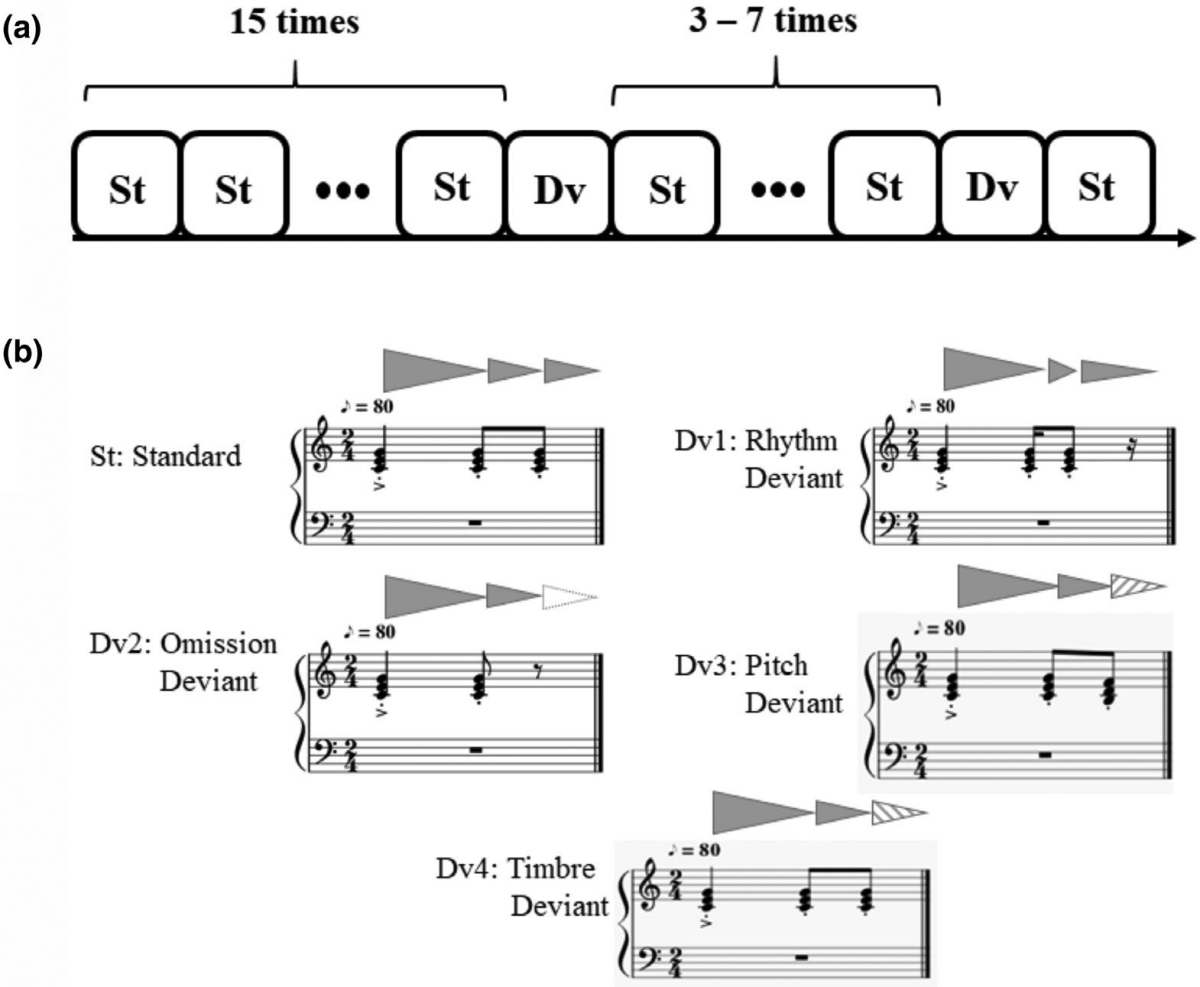
(a) The Experimental protocol. (b) The standard and deviant stimuli

The stimulus material consisted of an auditory rhythm in 2/4 meter on one chord that was presented repeatedly and continuously at 100 bpm with a piano sound. The standard rhythm consisted of a C Major chord (three tones, 261.6, 329.6, 392 Hz) with a duration of a quarter note (600 ms) at the beginning of each bar followed by two eighth note chords (300 ms, similar to the first chord in frequency content). The rhythmic changes (rhythm deviant) consisted of replacing the two eighth note chords with one sixteenth note chord (150 ms), one eighth note chord, and one sixteenth rest (Figure 1). The frequency content of the chords did not change either between the rhythm standard and deviant conditions, or between different trials. The omission deviant was created by silencing only the last chord of the standard condition (Figure S1). Analyzing a rhythmic sequence, one can imagine a "tree" structure, corresponding to the hierarchical representation of a sequence of timed events (notes in music). In this tree, the "root" node at the highest level of the hierarchy is considered as the whole bar, the nonterminal nodes signify the lower level metrical units, and the terminal nodes of the tree are all either (sounded) notes or rests (Longuet‐Higgins & Lee, 1984). Three rhythm trees are presented in Figure S1 showing the rhythmic structure of the standard stimulus and the rhythm and omission deviants. This figure shows how the rhythm deviant induces a new branch in the tree.

We distracted the participants from the main objective of the experiment by adding two other deviant conditions. The last chord was changed to a B diminished chord on three tones (246.9, 293.7, 349.2 Hz, Figure 1) to create a pitch deviant. Finally, we included a timbre deviant, for which one chord during the bar (randomly defined) was played by a violin sound. Subjects were instructed to watch a silent movie (March of the Penguins, Warner, ASIN B000BI5KV0) and press a button whenever they detected a timbre deviant (subjects recognized the timbre deviant with an accuracy of 97.4 ± 1.2%). The purpose of including the aforementioned two deviants was not to have the attentional focus of the subjects aimed toward the rhythm manipulations in the experiment.

Stimuli were constructed using open‐source MuseScore 2 software and exported as wav‐files. A dynamic accent of 25 percent above the general intensity was induced on the first beat of each stimulus (of the measure) to reinforce the perceived meter (Geiser et al., 2009). This accent is indicated by a ‘‘>’’ in Figure 1b, in which the stimuli and experimental protocol are depicted.

In the main experiment, the stimuli were delivered in the context of an oddball paradigm. The experimental session included high‐probability standard stimuli (*p* = 85%, 2,850 trials) interspersed with four infrequent deviant patterns, accounting for the remaining stimuli (*p* = 15%, 150 trials of the timbre deviant, and 105 trials for the rhythm, omission, and pitch deviants each). The choice of a slightly more frequent appearance of the timbre trials was to have a balanced distribution of distractors among standard and deviant trials (Koelsch et al., 2013). The order of the four deviant stimuli was pseudorandomized among the standard trials, enforcing three to seven standard stimuli between successive deviant trials. Control stimulus blocks, corresponding to rhythm and omission deviants, presented 200 times continuously without any standard trial in between, were used to evaluate the response to the rhythm and omission deviant trials in the main experiment. The presentation order for these blocks was random during the experimental protocol and counterbalanced among subjects, in order to avoid biasing the neural response by repetitive presentation of the trials in the control blocks at a specific time during the experiment. This strategy has been employed previously (Ladinig et al., 2009; Winkler et al., 2009), and assumes that repetitive representation of the standard stimuli after the control block resets the experiment conditi

on from the one of the control blocks to that of the main experiment.

We then performed a control experiment during which the rhythm deviant stimulus replaced the standard condition and the deviant condition consisted of silencing its last chord in the context of an oddball paradigm. This experiment was conducted to take the impact of the difference in the duration of the penultimate chord between the standard and rhythm deviant conditions into consideration, and allowed us to further explain the variation in the neural response due to the omission of the tone from that observed in the rhythm deviant condition. More precisely, in the main experiment paradigm, the expectation was violated 150 ms after the penultimate chord by earlier arrival of the last chord in the rhythm deviant, whereas the violation arrived 300 ms after the penultimate chord in the omission deviant by absence of the last expected chord (see Figure S1). Therefore, considering the latency of the neural response to the penultimate chord and the arrival times of the violations of expectation, the control experiment was required to address the role of the two different durations in modulation of the mismatch response characteristics (see SI for more details).

Stimuli were delivered through two speakers at 65 dB SPL using Psychtoolbox MATLAB. The total duration of the experiment was ~78 min. The neural response to the timbre deviants was not analyzed, whereas the response to the pitch deviants is reported and discussed in SI (see Figure S7 and S8).

### 2.3 EEG acquisition and preprocessing

High‐density EEG Data were acquired using a high impedance amplifier Net Amp 300 and Net Station 5 with a sensor net consisting of 128 electrodes (Geodesic Sensor Net, Electrical Geodesics, Inc., USA). Impedances were kept below 50 kΩ. The EEG was digitized at a 1,000‐Hz sampling rate, with a Cz vertex electrode as reference. The recorded signals were analyzed with MATLAB® software (The MathWorks, Inc., Natick, Massachusetts, United States), using FieldTrip (Oostenveld et al., 2011), EEGLAB (Delorme & Makeig, 2004), and custom MATLAB functions and codes. A two‐pass 0.5–100 Hz finite impulse response (FIR) bandpass filter (order = 3 cycles of the low‐frequency cut‐off) from the EEGLAB toolbox was applied to remove low and high‐frequency artifacts from the EEG signals. Artifact‐ridden channels were removed and interpolated. Also, a 50‐Hz notch filter was applied to remove the line noise. Artifacts (e.g., eye‐blink, eye‐movement, and muscle activity) were then removed by independent component analysis (ICA) using the EEGLAB toolbox. The preprocessed data were later epoched starting 650 ms before the onset of the deviant chord and ending 800 ms after. Epochs were excluded if the standard deviation of amplitude exceeded 25 μV within two moving windows of 200 and 800 ms or any sampling point exceeded 75 μV at any electrode location. EEG data were later re‐referenced to the average reference. After artifact rejection, the number of remaining trials for different conditions was 87.98 ± 13.47 (rhythm deviant), 176.58 ± 18.24 (rhythm control), 87.15 ± 14.78 (omission deviant), and 167.4 ± 23.93 (omission control).

### 2.4 Event‐related potential (ERP)

A 25‐Hz low‐pass FIR filter (13 cycles) was applied to calculate the ERP response. For each trial, zero was set at the onset of the third chord (the expected location of the third chord for the omission condition), with the baseline being set at −500 to −300 ms and −650 to −450 ms for the rhythm and omission deviants, respectively (for both conditions the baseline was set at 250–450 ms from the onset of the first chord, which was after the disappearance of the response to the first chord). Event‐related potentials were computed by averaging the EEG trace of the remaining trials for each condition after baseline correction. A nonparametric cluster‐based permutation procedure (5,000 permutations), implemented in the FieldTrip toolbox (Maris & Oostenveld, 2007), was applied to search for significant changes in the deviant condition relative to the control condition (see SI for details). The initial threshold for cluster definition and the minimum number of neighbors were set to *p* < .05 and four, respectively. Finally, the final significance threshold for summed *t* values within clusters was set to *p* < .05.

### 2.5 Time‐frequency representation (TFR)

TFRs were calculated per event epoch (‘mtmconvol’ function of the FieldTrip toolbox) for frequencies from 4 to 30 Hz (using Hanning tapers) and from 30 to 100 Hz (using discrete prolate spheroidal sequence tapers) in steps of 0.25 Hz. The TFR was calculated using a sliding window with a variable frequency‐dependent length that always comprised a full number of cycles (at least two cycles and an at least 100‐ms window length). Time‐locked TFRs of all epochs were then baseline corrected and averaged per participant. Statistical analysis was used to check for significant power changes corresponding to the deviant condition relative to the control condition. The cluster‐based permutation procedure (5,000 permutations), implemented in the FieldTrip toolbox, was applied to correct for multiple comparisons (see SI for details). The initial threshold for cluster definition and the minimum number of neighbors were set to *p* < .05 and four, respectively. Finally, the final significance threshold for summed *t* values within clusters was set to *p* < .05.

### 2.6 Phase amplitude coupling (PAC)

We applied a method introduced by (Tort et al., 2010) to simultaneously assess PAC for a large number of frequency pairs. For a given frequency pair, extracted 3.6‐s epochs (including three trials: the target trial, and the preceding and following trials)—sufficiently long to prevent any edge effects during filtering—were filtered in both frequency ranges. Lower frequencies ranged from 6 to 15 Hz (0.25‐Hz increments, the bandwidth was gradually increased from 0.75 to 1.875 Hz) and higher frequencies ranged from 60 to 100 Hz (0.25‐Hz increments, the bandwidth was gradually increased from 7.5 to 12.5 Hz). The time series of the lower frequency phase and the higher frequency amplitude were then extracted using the Hilbert transform. The deviant responses (the third chord for the rhythm deviant, and the expected interval of the third chord for the omission condition) were concatenated and the lower‐frequency phases binned into eighteen 20° bins spanning the [−π, π] interval and the corresponding mean amplitude of the higher frequency was computed for each phase bin and then normalized by dividing it by the sum over all bins. Next, the deviation of the PAC profile from a uniform distribution was quantified by defining the modulation index (MI) in terms of the Kullback‐Lieber distance between the amplitude distribution *P* and a uniform distribution *U*, $D_{\mathrm{KL}}\left(P,U\right)=\log\left(\mathrm{nbins}\right)‐H\left(P\right)$, where the Shannon entropy *H* of the distribution *P* is $H\left(P\right)=‐\sum_{bin=1}^{N}P\left(\mathrm{bin}\right)\times \text{log}\left[P\left(\mathrm{bin}\right)\right]$. Briefly, the MI of (Tort et al., 2010) specifically measures deviations from a uniform distribution; if the high‐frequency EEG mean amplitude shows no systematic relationship with the low‐frequency phase, the high‐frequency amplitude in each low‐frequency phase bin will tend toward the overall average high‐frequency amplitude, resulting in a flat or uniform distribution. The MI ranges from 0 to 1; a value of 0 shows that the mean amplitude is uniformly distributed over the phases and an MI of 1 shows that the mean amplitude has a Dirac‐like distribution.

$$MI=\frac{D_{\mathrm{KL}}\left(P,U\right)}{\log\left(\mathrm{nbins}\right)}$$

For statistical analysis, the cluster‐based permutation procedure, implemented in the FieldTrip toolbox, was used to compare the deviant comodulogram with that corresponding to the control data at the group level. The initial threshold for cluster definition and the minimum number of neighboring were set to *p* < .05 and four, respectively. The final threshold for significance of the summed *t* value within clusters was set to *p* < .05. In addition, surrogate chance level PAC data were created over the region of interest (as average over certain electrodes, based on the comparison between deviant and control conditions, see also Section 3.3) by random shuffling the phase time series (500 times) and then calculating the MI value between the shuffled phase time series and the original amplitude time series. We obtained a surrogate MI value for each frequency band and then subject over the ROI. Finally, the cluster‐based permutation procedure was used to compare the empirical MI with that corresponding to the surrogate data at the group level for rhythm deviant conditions in the main and control experiment. The initial threshold for cluster definition and the final threshold for significance of the summed *t* value within clusters were both set to *p* < .05.

We further investigated the nesting of theta‐gamma activity (6.5–8.5 Hz for the phase frequency and 61–78 Hz for the amplitude‐frequency, the choice of the phase/amplitude frequencies was made based on the results of the comodulogram analysis) in the time course of the deviant response by calculating the PAC over a sliding window of 200 ms in steps of 5 ms. We thus concatenated the 200‐ms windows corresponding to each trial (the epochs were cut after applying the filter and Hilbert transform) and performed the aforementioned method to calculate the MI. This procedure was repeated at each time step, which resulted in a MI time‐series with a resolution of 5 ms. For statistical analysis, the cluster‐based permutation procedure was implemented.

### 2.7 Statistical analyses

Normality of data distribution was verified by the Lilliefors test, which is an improved approach compared to the Kolmogorov test (Lilliefors, 1969). We examined the significance of ERP amplitude and latency between the rhythm deviant and omission conditions using a two‐tailed paired‐samples *t* test. Toward this, we calculated the peak and latency over a predefined time window and a fronto‐central region of interest (ROI, ERP amplitudes were computed from the average of the electrodes presented in Figure S4A), which was the same for both conditions. For time‐frequency statistics comparing rhythm and omission conditions, the power was defined as the maximum power over a specific time window (50–300 ms), averaged over the frequency range 4–10 Hz and over a predefined fronto‐central ROI (Figure S4B). The emergence time of the oscillatory activity was defined as the time when the power reached 30% of its maximum value. The time‐frequency characteristics (power and latency) were compared between the rhythm deviant and omission conditions using two‐tailed paired‐samples *t* test and Wilcoxon signed‐rank test. The relationship between high‐frequency power and P3a amplitude was investigated via Spearman correlation.

## 3 RESULTS

Our results, presented in detail below, show that both small and large rhythmic deviations induced a neural response with a time course and topographical distribution typical of MMNs. They further demonstrate a significant difference in the amplitude of the MMN between the two conditions. Interestingly, we only observed a P3a for the rhythm deviants, which followed the MMN and was concurrent with the emergence of gamma activity over the left frontal area. In this paper, the statistical analyses were based on cluster‐based permutation to deal with the high spatial, temporal and spectral dimension of the data and to correct for multiple comparisons. However, it needs to be taken into consideration that cluster‐based methods can underestimate the latency, spatial, or frequency extent of effects (Sassenhagen & Draschkow, 2019).

### 3.1 Event‐related potentials

**FIGURE 2:**
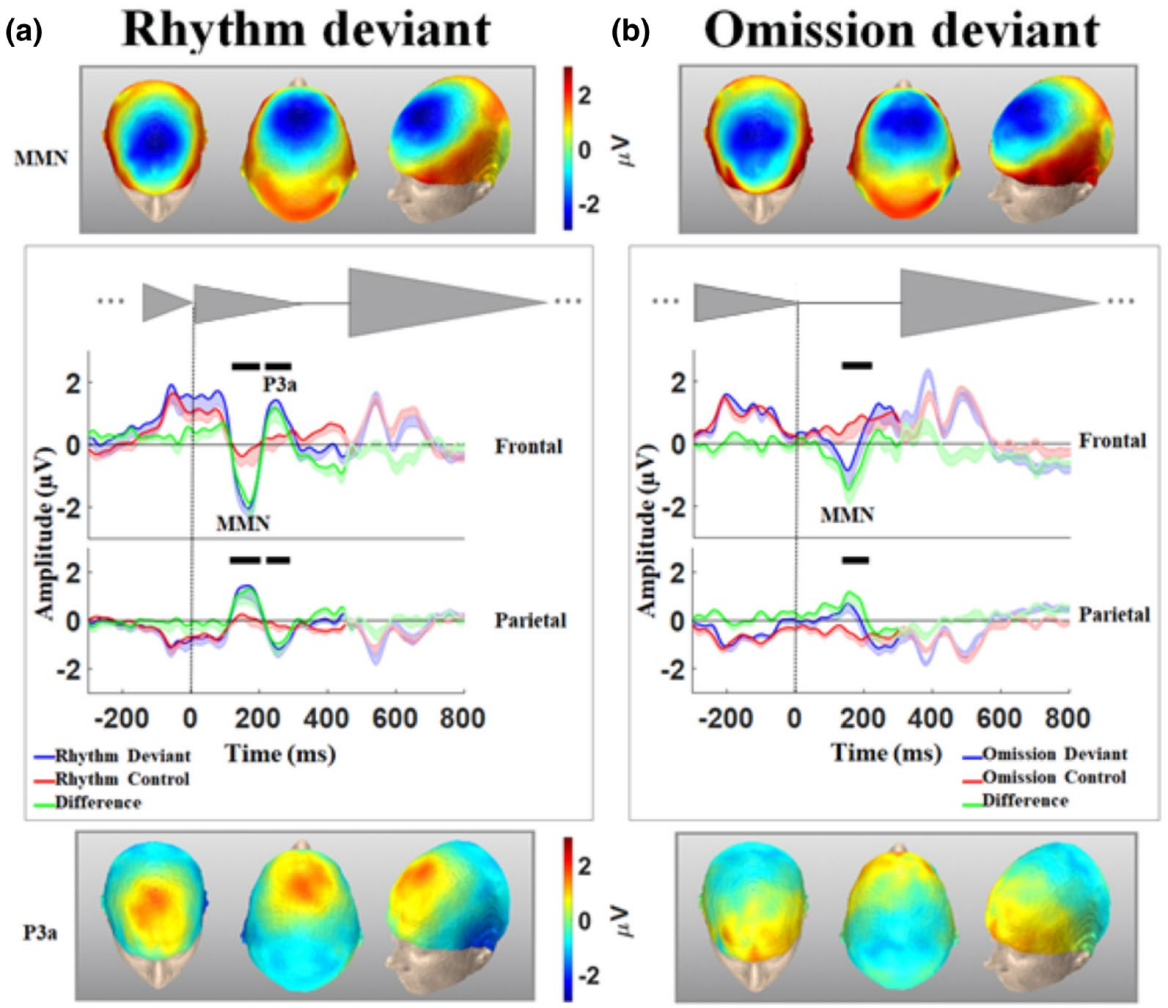
Event‐locked analysis of rhythm and omission conditions. The onset of the deviant chord was set to zero and the next trial started at 450 and 300 ms for the rhythm and omission conditions, respectively. For both conditions, the baseline was set to 250 to 450 ms from the onset of the first chord. (a) Grand average of ERP (‐SE) for the rhythm deviant condition, the control condition, and their difference over frontal and parietal clusters. (b) Same as (a) for the omission condition. Both rhythm and omission deviants elicited an MMN. However, a P3a followed the MMN for only the rhythm deviants. The black bars over the ERP figures represent the time intervals of significant difference between the deviant and control conditions (*p* < .05, corrected, marked according to cluster‐based permutation analysis). The topography of each significant time window is shown in the boxes: above for the MMN corresponding to the rhythm and omission deviant conditions and below for the P3a in the rhythm and omission (not significant) deviant condition

The ERP response to the rhythm and omission deviants are depicted in Figure 2a and b. We considered the baseline time window [−500 to −300 ms] for the rhythm and [−650 to −450 ms] for the omission condition (that is [250–450 ms] from the onset of the first chord in both conditions). The grand average ERPs showed enhanced early (~100–200 ms) frontal negativity, consistent with the typical time window of MMN, for both deviant conditions with respect to the control conditions (Figure 2). For the rhythm condition only, the MMN was followed by a subsequent positive deflection in the 200 to 300‐ms time window, indicative of a P3a (Figure 2a). Through visual inspection, both components were more pronounced over the frontal and fronto‐central electrodes and demonstrated an inverting polarity over the posterior electrodes.

Cluster‐based statistics revealed four spatio‐temporal clusters for the rhythm condition (their time intervals are specified by the thick black lines in Figure 2a): (1) a negative cluster (*p* = .0036, corrected) comprising frontal and fronto‐central electrodes and extending approximately over 120–202‐ms post‐final chord, (2) a temporo‐posterior positive cluster (*p* = .0008, corrected) synchronous with the first cluster, 114–204‐ms post‐final chord, (3) a negative posterior cluster (*p* = .0376, corrected) extending approximately over 221–290‐ms post‐final chord, and (4) a positive frontal cluster (*p* = .0328, corrected) synchronous with the third cluster, 217–294‐ms post‐final chord. Spearman correlation analysis revealed a significant correlation between the MMN amplitude, averaged over 130–180 ms, and the P3a amplitude, averaged over 200–300 ms (*r* = 0.8909, *p* = .0014), over the fronto‐central cluster. Cluster‐based statistics revealed two spatio‐temporal clusters for the omission condition (their time intervals are specified by the thick black line in Figure 2b): (1) a negative cluster (*p* = .0004, corrected) comprising frontal and fronto‐central electrodes and extending approximately over 133–221‐ms post‐final chord and (2) a posterior positive cluster (*p* = .0112, corrected) synchronous with the first cluster, 137–215‐ms post‐final chord. The topographical distribution of the clusters corresponding to different time windows in the course of the deviant response is shown in supplementary Figure S2 (electrodes belonging to each cluster are presented in Figure S3). In addition, the supplementary movie illustrates the evolution of the ERP response over the scalp during the course of the deviant response for both rhythm and omission conditions.

The MMN amplitude (considered as the peak over a fronto‐central ROI, Figure S4 A, over 100–250 ms), corresponding to the rhythm condition, was significantly larger than that of the omission condition, as shown by a paired sample *t* test (*t* = 2.4214, *p* = .0339). There was no significant difference between the latency of MMN corresponding to the two conditions (*t* = 1.6769, *p* = .1217). We also performed a control experiment during which the rhythm deviant stimulus replaced the standard condition in the context of an oddball paradigm and the deviant condition consisted of silencing its last chord. The aim of this experiment was to show that having the omission MMN being smaller compared to the mismatch corresponding to the rhythm deviant is not related to the relative distance of the chord being omitted to the penultimate chord (see SI for more details). During this control experiment, omission of the last chord in Dv1 (Figure S1) resulted in a smaller MMN than the rhythm deviant response in the main experiment and it was not followed by the significant P3a that was observed in the rhythm deviant condition during the main experiment (Figure S5a). The results of this control study confirm that the omission condition results in a relatively smaller MMN and does not elicit a significant P3a component. These results support the results corresponding to the main experiment and the difference observed between the omission and rhythm deviant conditions.

### 3.2 Time‐frequency representation

**FIGURE 3:**
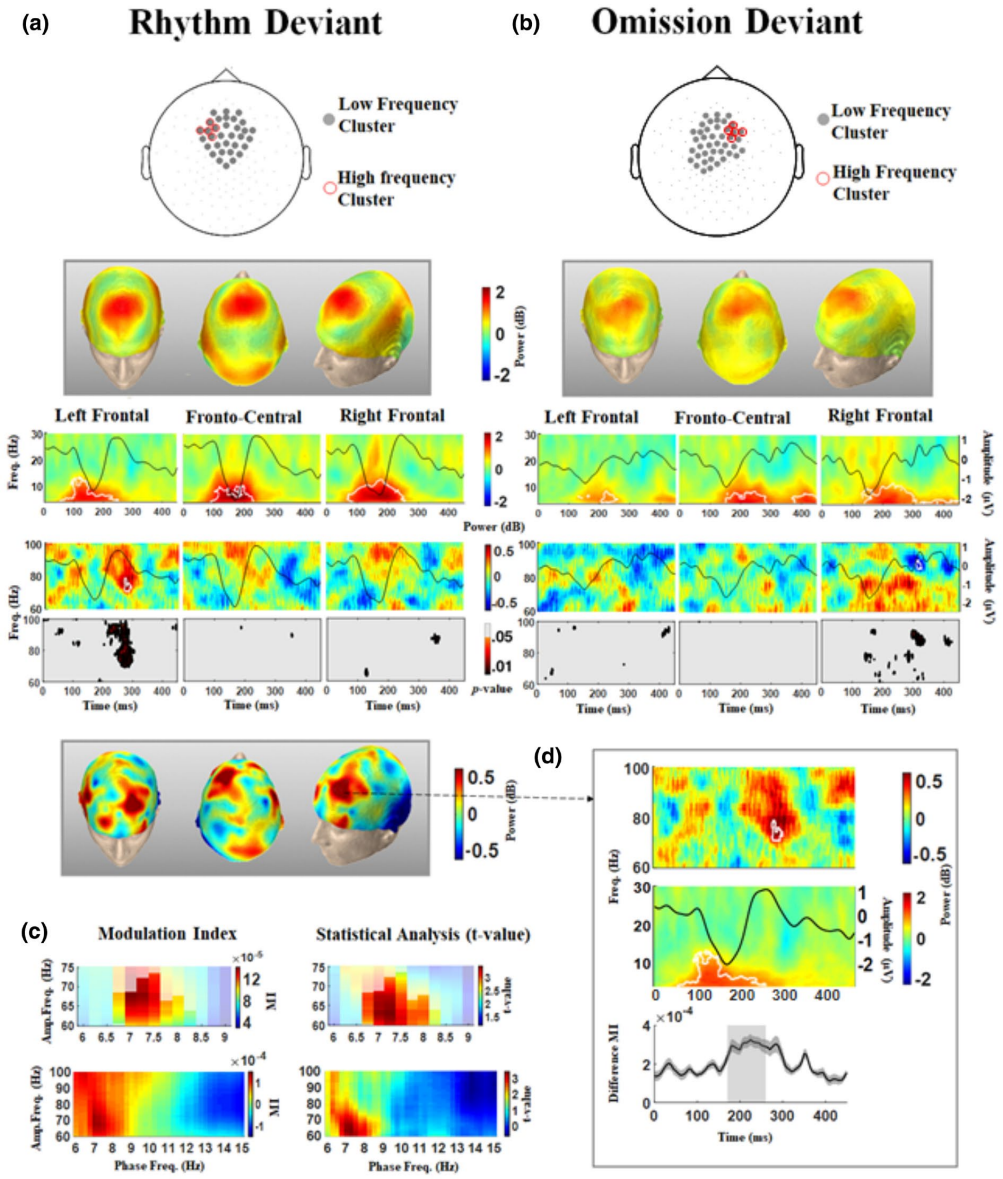
Event‐locked analysis of the rhythm and omission deviant conditions. The average TFR locked to the beginning of the rhythm deviant (a) and omission deviant (b) trials. The corresponding ERP of the ROI is superimposed on each TFR to better illustrate the results. The statistically significant changes from the control condition are indicated by a white contour. Rhythm deviant: low‐frequency cluster: 90 to 228 ms, *p* = .0008, corrected; high‐frequency cluster: 260 to 289 ms *p* = .0142, corrected. Omission deviant: low‐frequency cluster: 140 to 283 ms, *p* = .0002, corrected; high‐frequency cluster: 307 to 330 ms, *p* = .048, corrected. The figures below the high‐frequency TFRs show the uncorrected *p* values corresponding to the comparison between the deviant and control conditions (paired‐sample *t* test). The topographical distributions of the electrodes belonging to the significant low‐ and high‐frequency clusters are specified on the head map on top. The topographical distribution of the average power over the frequency and time window corresponding to each cluster is presented in the boxes (above for the low‐frequency and below for the high‐frequency TFR). (c) Comodulograms of phase‐amplitude coupling analysis over the 450‐ms window corresponding to the rhythm deviant condition. Comparison of the rhythm deviant condition with the control condition showed a single cluster with significant MI (*p* = .0318, corrected). (d) The difference between the rhythm deviant and control conditions in low‐frequency TFR, high‐frequency TFR, and time‐varying PAC over the time course of the rhythm deviant condition is shown. The significant cluster observed when comparing the two conditions is marked in all the three figures. The time‐varying PAC is presented as the mean ± SE

For each participant, we calculated the TFR over 4 to 30 Hz and 60 to 100 Hz and set the zero to the onset of the deviant condition to determine the power modulation during the time course of the deviant response (Figure 3). The mean TFRs over the left, middle, and right frontal electrodes were computed to show the spatial dynamic of power fluctuations in the low‐ (4–30 Hz) and high‐frequency (60–100 Hz) ranges for the rhythm and omission conditions (deviant minus control), respectively (Figure 3a and b).

As illustrated, the pronounced oscillatory power over the 4 to 10‐Hz frequency range coincided with the temporal location of MMN, in agreement with the modulation of low‐frequency power during ERP components (Fuentemilla et al., 2008; Kaser et al., 2013; Pearce et al., 2010). Cluster‐based statistics revealed a spatio‐temporal cluster over 90 to 228 ms (*p* = .0008, corrected) for the rhythm condition and over 140 to 283 ms (*p* = .0002, corrected) for the omission condition over the 4‐ to 10‐Hz frequency range, comprising the frontal and frontocentral electrodes (clusters are presented in Figure 3). The power corresponding to the rhythm condition was larger than the omission condition (calculated as the peak value between 50 and 300 ms of the averaged frequency response between 4 and 10 Hz), however the difference failed to reach statistical significance (*t* = 1.779, *p* = .1028, paired‐sample *t* test). There was no significant difference between the emergence times of the low‐frequency response corresponding to the two conditions (*p* = .0957, Wilcoxon signed‐rank test). However, the latency of the power corresponding to the omission condition was significantly larger than the rhythm condition (*t* = 3.0506, *p* = .0110, paired‐sample *t* test).

In the rhythm deviant trials, TFR analysis over the 70‐ to 100‐Hz frequency range (Figure 3a) showed pronounced high‐frequency oscillatory power over the left frontal electrodes. The significant difference relative to the control condition was restricted to the 260 to 289‐ms time window (*p* = .0142, corrected) after the onset of the deviant chord (Figure 3a), concurrent with the descending slope of the P3a component. Interestingly, the high‐frequency oscillatory power, as averaged between 200 and 300 ms, correlated with the P3a amplitude over the left frontal cluster (*r* = 0.6909, *p* = .0231), as revealed by Spearman correlation analysis.

In the omission deviant trials, TFR analysis over the 60‐ to 80‐Hz frequency range (Figure 3b) showed a non‐significant increase in high‐frequency oscillatory power over the right frontal electrodes between 100 and 300 ms, followed by a significant decrease comprising five right frontal electrodes and extending approximately from 307 to 330 ms (*p* = .048, corrected, Figure 3b), which coincided with the onset of the next standard trial.

### 3.3 Phase amplitude coupling

To further address the underlying mechanisms of rhythm perception and investigate the inter‐relationships of oscillatory activities during the processing of rhythm deviations, we evaluated PAC across a broader frequency range by applying the comodulogram analysis (Tort et al., 2010). The modulation index (MI) reflects the degree to which the amplitude of the higher (modulated) frequency varies as a function of the phase of the lower (modulating) frequency. We performed the PAC analysis over all electrodes for both deviant conditions, with the phase frequency ranging from 6 to 15 Hz and the amplitude frequency ranging from 60 to 100 Hz, as explained in Materials and Methods. Cluster‐based statistics revealed a significant cluster (*p* = .0318, corrected) when comparing the 450‐ms window between the rhythm deviant condition with the control condition. This cluster demonstrated the presence of significant PAC for the rhythm deviant condition, in which the power of the 60‐ to 75‐Hz frequency range was modulated by the phase of the 6.5‐ to 8.5‐Hz frequency range (Figure 3c). In addition, we assessed the statistical significance of the observed CFC by comparing the results with those generated with the shuffled surrogate data—the same data as for the original PAC analysis, and with exactly the same spectral power characteristics. This was carried out over the average of electrodes over the left frontal region, leading to a significant cluster in the comparison performed over the PAC between rhythm deviant and control conditions. This procedure showed a single positive cluster (*p* = .002, corrected, Figure S6). This cluster corresponded to the theta‐gamma PAC. The same procedure was carried out over the same region of interest, but for the data corresponding to the rhythm deviant condition in the follow‐up control experiment (conducted over a different group of participants, see also the SI). Over the same region of interest, the procedure led to a single positive cluster (*p* = .023, Figure S6), however, the strength of the theta‐gamma PAC was smaller compared to the theta‐gamma coupling observed during the main experiment. The different number of participants in the two experiments as well as the variations in the experiment design might have played a role in the differences observed between the two aforementioned analyses. Together, the two comparisons showed that the pronounced theta‐gamma coupling in the rhythm deviant condition was significantly stronger from both a random non‐significant condition (surrogate data), and the control condition. There was no significant cluster, when comparing the omission deviant and control conditions.

We further investigated the temporal pattern of PAC during the time course of the deviant response. The mean time‐varying PAC over subjects for the rhythm deviant condition is illustrated in Figure 3d. The plotted MI is the time‐varying deviant MI minus the time‐varying control MI over 6.5 to 8.5 Hz for the phase frequency and 61 to 78 Hz for the amplitude‐frequency. The difference between the two conditions was significant and increased over a time window from 170 to 260 ms (*p* = .0459, corrected) from the onset of the deviant chord. Interestingly, the timing of the PAC for the deviant condition was concurrent with the late parts of the low‐frequency TFR cluster and coincided with the significant high‐frequency TFR cluster. These PAC results not only corroborate the main findings from our event‐based analysis but also highlight that the observed late high‐frequency oscillatory activity in the TFR analysis was nested in the low‐frequency oscillations in the theta range. There was no significant difference between the PAC corresponding to the omission deviant condition compared to the control condition in the time course of the deviant response.

## 4 DISCUSSION

Both rhythm and omission deviations induced a typical MMN, similar in time course and topographical distribution, with a significantly higher amplitude for rhythm deviations. In addition, a significant P3a was elicited only for the rhythm deviant. Furthermore, rhythm violation through modulation of the rhythmic structure elicited significant late gamma‐band activity over the left superior frontal area, which occurred concomitantly with the P3a component. This gamma oscillation was nested in theta oscillations, resulting in significant phase‐amplitude coupling. The power of the gamma oscillation correlated with the amplitude of the P3a component over the same ROI for the rhythmic deviant condition only. Omission of the last chord in the rhythmic sequence also elicited an MMN, but this component was not followed by a later P3a‐positive component in the left frontal area and did not elicit a significant gamma‐band response.

Rhythm perception consists of extracting regularities from the sound stream and shaping temporal expectations about the future events. It is considered to be a Bayesian process (Elliott et al., 2014; Koelsch et al., 2019; Lumaca et al., 2019), which fits with the framework of predictive coding (Friston, 2005). Here, we presented two types of deviant conditions: rhythm and omission, in which the former condition was created by the last chord arriving earlier than expected and the latter by omission of the last chord. Exposure to the rhythmic deviant condition changes the “tree” structure, with the addition of a new branch, which requires updating of the neural model. Conversely, omission of the last chord maintains the tree structure and therefore results in a relatively smaller violation than the rhythmic violation and a relatively smaller prediction error, which might not require modulation of the neural model. Studies of the neural correlates of novelty responses show that a significant MMN is elicited by both small and large deviants, whereas a significant P3a is elicited only by large deviants (Friedman et al., 2001). It has been shown that MMN is sensitive to sensory or lower‐level violations, reflecting the detection of deviant events, whereas the later responses arrive only when there is a higher‐level violation of the regularity of the underlying structure (Chennu et al., 2013; Wacongne et al., 2011). The P3a component is associated with the evaluation of the deviant events (Lumaca et al., 2019) and reflects updating of the prediction model, which involves a broad fronto‐parietal network (Wacongne et al., 2011). The P3a has been linked to musical expectancy, being sensitive to large violations of rhythmic (Vuust et al., 2009), metric (Jongsma et al., 2004), melodic (Trainor et al., 2002), and harmonic (Janata, 1995) structure. In our study and during first‐level processing, the MMN was sensitive to the prediction error being larger for the rhythm deviant than the omission deviant, for which the rhythmic tree structure was changed. We suggest that violation of the rhythmic tree structure in the rhythm deviant elicited a larger MMN, which moved to higher areas in the chain of auditory processing, leading to integration with the higher areas and then updating of the predictive model of the rhythmic structure in the higher levels of the hierarchy. This hypothesis is supported by the (*i*) occurrence of the P3a component, the amplitude of which correlated with that of the MMN, and (*ii*) by the gamma‐band activity nested in the P3a component, with its power being correlated with the P3a amplitude. It has to be noted however that it was not necessarily the specific new metrical level used in this study (induced by the rhythm deviant) that elicited the neural response. Other large enough violations of the rhythmic structure, by other degrees of changes in chord durations could have elicited similar neural responses.

The observed late induced gamma activity in response to the rhythm deviant condition, in contrast to the control condition, reflects focal synchronized neural activity (in contrast to observed earlier wide‐spread effects) at the left frontal electrodes (Figure 3). Gamma‐band activity is investigated in processes related to the auditory system and is suggested to reflect attention, anticipation, and expectation (Snyder & Large, 2005; Sokolov et al., 2004; Zanto et al., 2005); (Bhattacharya et al., 2001). Induced “late” gamma activity, which typically emerges later than 200 ms, even in concomitance with the P3 component (Başar‐Eroglu & Başar, 1991), is suggested to be a signature of processes such as response selection or context updating (Herrmann et al., 2004). During the creation of a phonetic mismatch response, induced gamma activity (84–88 Hz) follows the evoked mismatch response by 130 ms over the left inferior frontal cortex (Kaiser et al., 2002). Focal increased gamma activity (50–90 Hz) has also been observed over the left superior frontal area in response to an acoustic mismatch in the context of an oddball audiovisual paradigm, suggested to reflect higher‐order auditory functions following the mismatch response (Kaiser et al., 2003, 2005). It has been proposed that the late gamma activity is specifically related to the match between stimulus‐related information and top‐down factors, as well as the emergence of an object representation (Noesselt et al., 2003; Tallon‐Baudry & Bertrand, 1999). We suggest that the observed induced late gamma‐band activity in this study reflects the integration of bottom‐up and top‐down processing toward refining the predictions of the neural model corresponding to the temporal structure of the events in higher‐level cortical areas.

Although, the observed gamma activity can be interpreted as a neural correlate of rhythm deviation, the results have to be interpreted with caution, considering the small population size. In addition, the study design permitted the comparison only between the omission and rhythm conditions. Future work must manipulate the attentional state of the subjects as well as the degree of deviation from the rhythm structure and investigate high‐frequency power modulation and CFC as a function of the deviations.

Recent studies in humans using ECoG, EEG, MEG, and fMRI have demonstrated that local error signals are restricted to the primary auditory cortex, whereas error signals corresponding to the violation of the global structure propagate to distributed areas in the frontal cortex (Bekinschtein et al., 2009; Chennu et al., 2013; El Karoui et al., 2015; Wacongne et al., 2011). The frontal cortex encodes the global and abstract characteristics of a sequence (Dehaene et al., 2015; Wang et al., 2015). Signals reflecting the update of the neural model are primarily found in the prefrontal cortex and dorsolateral prefrontal cortex, areas important for working memory‐related processing (Curtis & D'Esposito, 2003; Gilbert & Kesner, 2006). Chao et al. suggested that these brain structures “generate and hold an internal representation of the entire sequence of stimuli” and therefore can later generate error signals when an unexpected novel sequence is heard (Chao et al., 2018). We suggest that the elicited gamma‐band activity over the left frontal cortex may reflect the underlying mechanisms involved in updating of the neural model of the entire rhythmic structure over the frontal areas.

An interpretation for the increased focalized PAC in our results may be that the lower‐frequency theta oscillations synchronized the synaptic input toward refinement of the predictive model of the temporal pattern of the music structure, which elicited the local high‐frequency gamma activity at the time of the P3a component. However, this hypothesis is speculative at present and requires further studies. The gamma‐band activity was nested in the late theta activity. Although the low‐frequency oscillatory activity was observed for both the omission and rhythm conditions, the elicited nested gamma activity and significant PAC were only observed for the rhythm deviant. Interestingly, in agreement with the observed gamma‐band activity, the PAC index became significant only at the time of the emergence of the gamma‐band activity and in coalescence with the P3a component. PAC is a potentially useful measure of coupling between neural oscillations on different timescales. The mechanisms underlying PAC have recently received much attention in both experimental and theoretical studies. It has been suggested that PAC supports the encoding, storage, and retrieval of information (Bergmann & Born, 2018; Fell & Axmacher, 2011). PAC translates as precise temporal relationships between modulating and modulated frequencies in (for example) the thalamocortical and hippocampal networks during sleep (Staresina et al., 2015), in the hippocampus during the operation of multi‐item working memory (Axmacher et al., 2010), and in the cortical networks during cognitive functions (Canolty et al., 2006; Chacko et al., 2018; Combrisson et al., 2017). It has been hypothesized that the phase of the slower oscillation generally reflects greater excitability among postsynaptic neurons, which in turn synchronizes the synaptic input (as reflected by an increase in the amplitude of the faster oscillation) (Bergmann & Born, 2018). The precise PAC between the theta oscillations and the elicited gamma‐band activity may reflect local spiking activity, which probably occurs for the revision of the predictive model developed in the higher levels of the hierarchy, which is locked to the phase of the slow theta oscillations, during the period in which the excitability of the neural population is higher, hence signaling the time window for updating the model. Further studies to address the information flow between the cortical structures are required to confirm this hypothesis.

Investigating the neural response to omission of tones is of specific interest in the framework of predictive coding, since it reflects an elicited response to violation of a sequence without any feedforward propagation of a sensory input (Bekinschtein et al., 2009), and therefore the neural response can be considered to reflect pure prediction (Chennu et al., 2016; SanMiguel et al., 2013). A recent study by (Chennu et al., 2013) showed that the elicited mismatch response can be best explained when assuming top‐down driven inputs in the dynamic causal modeling in higher‐order cortical areas. Interestingly, it has been demonstrated that evoked omission responses are sensitive not only to the timing of the stimulus, but also to its predicted identity (Auksztulewicz et al., 2018). Our results on the neural correlates of the omission response do not contradict previous findings related to the omission response. In the previous studies both the deviant and omission stimuli involved manipulation at the same hierarchical level of the stimulus structure (Chennu et al., 2013, 2016; Wacongne et al., 2011), which made the comparison between the presence and absence of a feedforward input feasible. In our study however, the omission or manipulation of the last chord affected the rhythmic structure differently. As the omission deviant was delivered in the context of an oddball paradigm and was only different in the absence of the last chord, the elicited MMN reflects an error, which was not proceeded by later ERP, and oscillatory activities that reflect a model update.

We presented the results corresponding to pitch deviants in the SI, where as illustrated the mismatch was not followed by either a P3a component, or significant high‐frequency oscillatory responses. The authors do not intent to suggest that the observed effect is exclusive to rhythm deviations. The results presented for pitch deviant have to be considered with caution, since a conclusion on the lack of the aforementioned effects cannot be made without studying the modulatory role of stimuli characteristics and paradigm design.

Considering the population size of the study, the results of the exploratory statistical tests have to be considered with caution. In addition, the design of the follow‐up control study did not allow the repetition of all the analyses (in terms of comparisons between deviant and control conditions). Even though comparisons with the surrogate condition were performed for both the main and control experiments, this has to be replicated in further control studies with the same experimental conditions. Future experiments in a large number of subjects can lead to more confidence regarding the role of high‐frequency responses in terms of both modulatory power and PAC, in predictive coding and its relationship with the P3a component. In the experiment design the instrumental piano tones were used for creating the stimuli. Although the chords faded out after the initial chord attack, the neural response might be impacted by the modulation of the duration of the penultimate chord. This is a factor that cannot be ruled out in the current context of the experiment design. The computed MI that explains the degree of coupling between different oscillatory activities of different frequency depends on different factors, including the technique that is used for quantification of this phenomenon (Tort et al., 2010), the neural signals that are being analyzed (e.g. EEG versus local field potentials that can lead to a better quantification of the coupling between oscillators, depending on the underlying mechanisms and neural structures), and the neural activity that is being studied (e.g. a cognitive task versus coupling in the thalamocortical networks during sleep). All these factors can lead to different MIs that define the degree of CFC and demonstrate the necessity for comparison of the observed effect with suitable control conditions or surrogate data.

This study addressed the neural oscillatory activity underlying rhythm processing. The results also proposed mechanisms that can be involved in predictive coding in terms of how the predictive error signal is processed and how the internal model is updated when confronting an input that violates the abstracted regularities. An interesting addition to this study which can be realized in future studies would be to address the role of the precision of predictions (Koelsch et al., 2019) in modulation of the high‐frequency oscillatory activity, observed presumably during the update of the model. Future studies need to be conducted to take into account the complexity of the temporal patterns (and as a result the assigned precision), which might modulate the prediction error and its processing. Further studies are required to address how this mechanism functions in processing temporal structures through other sensory modalities and the causal interactions in the neural networks that give rise to the observed activity. In addition, it has been shown that newborn infants develop expectation for the onset of rhythmic cycles and create a mismatch response to omission of the downbeat. An interesting question is how the mechanisms involved in the predictive coding of temporal structures, which are widely acknowledged to be an important feature of both music and language, evolve in the course of development and what are the differences between adults and newborns in terms of the mechanisms involved in creating a mismatch response.

## CONFLICT OF INTEREST

None.

## AUTHOR CONTRIBUTIONS

**Mohammadreza Edalati:** Formal analysis; Investigation; Methodology; Writing‐original draft; Writing‐review & editing. **Mahdi Mahmoudzadeh:** Conceptualization; Formal analysis; Investigation; Methodology; Project administration; Supervision; Validation; Visualization; Writing‐original draft; Writing‐review & editing. **Javad Safaie:** Funding acquisition; Project administration; Supervision; Writing‐review & editing. **Fabrice Wallois:** Conceptualization; Funding acquisition; Investigation; Methodology; Project administration; Resources; Supervision; Validation; Writing‐original draft; Writing‐review & editing. **Sahar Moghimi:** Conceptualization; Formal analysis; Funding acquisition; Methodology; Project administration; Supervision; Validation; Writing‐original draft; Writing‐review & editing.

## Supporting information


**FIGURE S1** Rhythm tree and the corresponding events for the three standard (St), rhythm deviant (Dv1), and omission deviant (Dv2) conditions. The green lines represent the position of the chords corresponding to different conditions. The corresponding sound waveform is presented below each condition (values are in seconds). Sound waveforms are presented as supplementary material (sound.wav)
**FIGURE S2** Topographical distribution of the clusters. Columns correspond to each time window from 75 to 300 ms and rows correspond to the ERP, low‐frequency TFR, and high‐frequency TFR, respectively. (A) Topographical distribution of the clusters corresponding to the rhythm deviant. (B) Topographical distribution of the clusters corresponding to the omission deviant
**FIGURE S3** Rhythm and omission ERP significant clusters. (A) MMN frontal and temporo‐posterior clusters of rhythm deviant. (B) P3a frontal and posterior clusters of rhythm deviant. (C) MMN frontal and posterior clusters of omission deviant
**FIGURE S4** Electrodes selected for additional ERP (A), and TFR (B) statistics analysis
**FIGURE S5** Event‐locked analysis of control blocks. (A) Event‐locked analysis of rhythm and omission II conditions. The onset of the deviant chord was set to zero and the next trial started at 450 ms. For all conditions, the baseline was set to 250 to 450 ms from the onset of the first chord. In the follow‐up control experiment, the rhythm deviant condition again elicited an MMN response followed by a P3a component. However, the omission II deviant only elicited an MMN response with its timing matching the MMN of rhythm deviant, without a P3a component. (B) Event‐locked analysis of each block separately. The onset of the deviant chord was set to zero and the next chord started at 450 ms for rhythm deviant, omission II deviant, and rhythm as standard condition, and at 300 ms for omission I and standard conditions
**FIGURE S6** Cluster‐based permutation results on phase‐amplitude coupling over the 450‐ms window, comparing the rhythm deviant condition in the main (*p* = .002, corrected) (A), and control (*p* = .023, corrected) (B) experiment with shuffled surrogate data. The grey regions correspond to frequency pairs for which the permutation analysis did not show significant PAC
**FIGURE S7** Grand average of ERP (‐SE) for the pitch deviant condition, the control condition, and their difference over frontal and parietal clusters. The onset of the deviant chord was set to zero and the next trial started at 300 ms. For this condition, the baseline was set to 250 to 450 ms from the onset of the first chord. The black bars over the ERP figures represent the time intervals of significant difference between the deviant and control conditions (*p* = 002 for frontal cluster and *p* = .004 for the posterior cluster, marked according to cluster‐based permutation analysis). The topography of significant time window is shown in the box
**FIGURE S8** Event‐locked analysis of the pitch condition. The average TFR locked to the beginning of the pitch deviant. The corresponding ERP of the ROI is superimposed on each TFR to better illustrate the results. A white contour indicates the statistically significant changes from the control condition. Low‐frequency cluster: 98 to 336 ms, *p* = .001, corrected. The figures below the high‐frequency TFRs show the uncorrected p values corresponding to the comparison between the deviant and control conditions (paired‐sample t‐test). The electrodes' topographical distributions belonging to the significant low‐frequency cluster are specified on the head map on top. The topographical distribution of the average power over the frequency and time window corresponding to each cluster is presented in the box

Video S1

Audio S1

Audio S2

Audio S3

Audio S4

Audio S5
