# Supplementary material for: Violation of rhythmic expectancies can elicit late frontal gamma activity nested in theta oscillations
Source: Psychophysiology. 2021 Jul 26;58(11):e13909. doi: 10.1111/psyp.13909 (PMC9285090; doi:10.1111/psyp.13909)
Supplement: Supplementary file 1 — FIGURE S1 Rhythm tree and the corresponding events for the three standard (St), rhythm deviant (Dv1), and omission deviant (Dv2) conditions. The green lines represent the position of the chords corresponding to different conditions. The corresponding sound waveform is presented below each condition (values are in seconds). Sound waveforms are presented as supplementary material (sound.wav) FIGURE S2 Topographical distribution of the clusters. Columns correspond to each time window from 75 to 300 ms and rows correspond to the ERP, low‐frequency TFR, and high‐frequency TFR, respectively. (A) Topographical distribution of the clusters corresponding to the rhythm deviant. (B) Topographical distribution of the clusters corresponding to the omission deviant FIGURE S3 Rhythm and omission ERP significant clusters. (A) MMN frontal and temporo‐posterior clusters of rhythm deviant. (B) P3a frontal and posterior clusters of rhythm deviant. (C) MMN frontal and posterior clusters of omission deviant FIGURE S4 Electrodes selected for additional ERP (A), and TFR (B) statistics analysis FIGURE S5 Event‐locked analysis of control blocks. (A) Event‐locked analysis of rhythm and omission II conditions. The onset of the deviant chord was set to zero and the next trial started at 450 ms. For all conditions, the baseline was set to 250 to 450 ms from the onset of the first chord. In the follow‐up control experiment, the rhythm deviant condition again elicited an MMN response followed by a P3a component. However, the omission II deviant only elicited an MMN response with its timing matching the MMN of rhythm deviant, without a P3a component. (B) Event‐locked analysis of each block separately. The onset of the deviant chord was set to zero and the next chord started at 450 ms for rhythm deviant, omission II deviant, and rhythm as standard condition, and at 300 ms for omission I and standard conditions FIGURE S6 Cluster‐based permutation results on phase‐amplitude coupling over the 450‐ms wi [file PSYP-58-0-s007.docx]

**Supplementary Information**


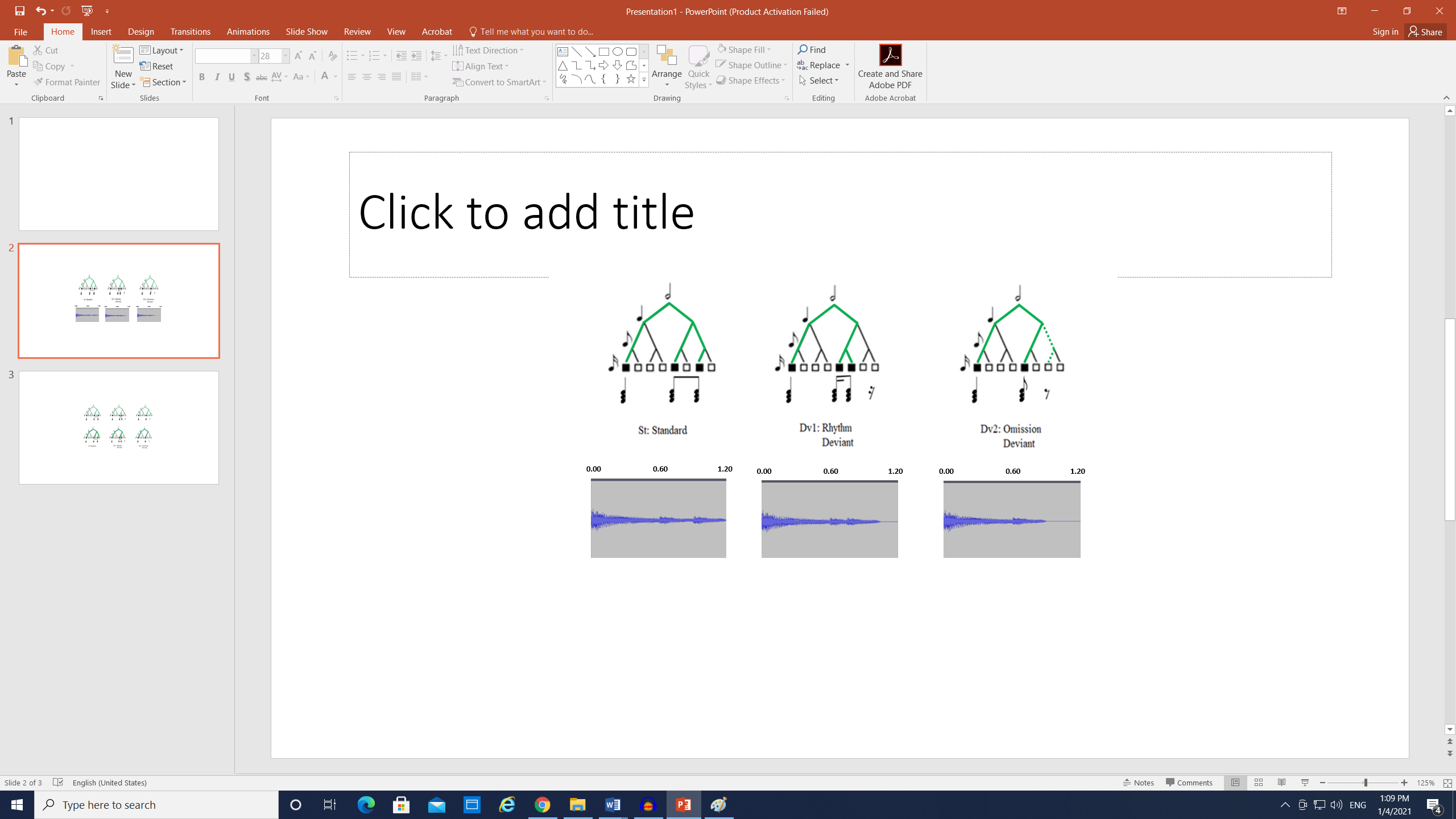


**Figure S1. Rhythm tree and the corresponding events for the three standard (St), rhythm deviant (Dv1), and omission deviant (Dv2) conditions. The green lines represent the position of the chords corresponding to different conditions. The corresponding sound waveform is presented below each condition (values are in seconds). Sound waveforms are presented as supplementary material (sound.wav).**

We asked the subjects to click a key when they hear the timbre deviant. The subjects recognized the timbre deviant by the accuracy of 97.4±1.2% on average.

**Statistical analysis of event-related potentials and time-frequency representations**

Considering the high spatial resolution of the EEG data and dealing with both the time and frequency variables, we did not make any a-priori assumption regarding the extent of the effects and also did not choose any specific band or ROI a-priori for statistical analyses. In order to compare the conditions during the ERP and TFR analyses, a nonparametric cluster-based permutation procedure, implemented in the FieldTrip toolbox, was applied to search for significant changes in the deviant condition relative to the control condition. After computing the *t*-value of a comparison between standard and deviant conditions in each channel and for each time-point (or time-frequency point for TFR), a spatio-temporal (spatio-temporo-spectral) cluster was defined as the sum of all *t*-values exceeding a limit set at .05, 2-tailed, in the data points closely related in space and time (and also frequency for TFR). A similar procedure was performed for each of 5000 random permutations of the conditions in order to establish the null distribution of cluster values. In each permutation analysis, the standard and the deviant condition data were randomly reordered across subjects. *t*-tests (*p*-value < .05) were then calculated for all electrode sites (128 electrodes) and each time point for ERP and each time-frequency point for TFR, to identify electrodes with a significant difference between the reordered data. Clusters were identified by considering only those (at least four) contiguous electrode sites with a *p*-value < .05, and the sum of the *t*-values was recovered for each cluster. This procedure was performed to obtain the null distribution from the data and to compute the corrected *p*-values.


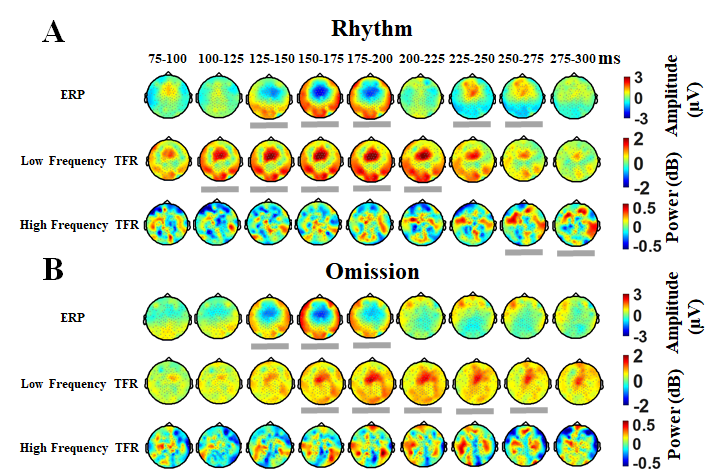
**The topographical distribution of the clusters**

**Figure S2. Topographical distribution of the clusters. Columns correspond to each time window from 75 to 300 ms and rows correspond to the ERP, low-frequency TFR, and high-frequency TFR, respectively. (A) Topographical distribution of the clusters corresponding to the rhythm deviant. (B) Topographical distribution of the clusters corresponding to the omission deviant.**

**
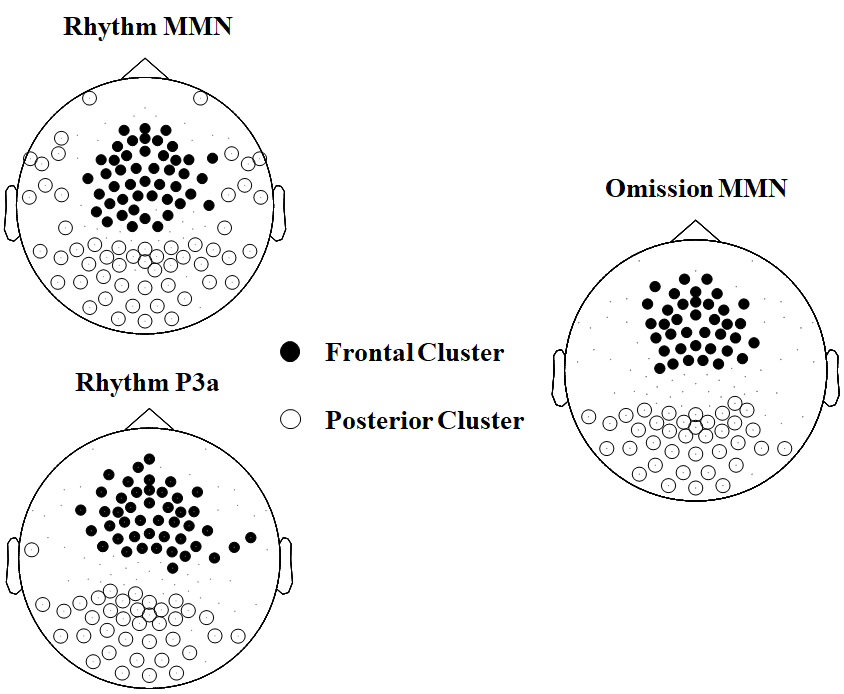
Rhythm and omission ERP significant clusters**

**C**

**B**

**A**

**Figure S3. Rhythm and omission ERP significant clusters. (A) MMN frontal and temporo-posterior clusters of rhythm deviant. (B) P3a frontal and posterior clusters of rhythm deviant. (C) MMN frontal and posterior clusters of omission deviant.**

**Electrodes selected for additional ERP and TFR statistical analysis**

**
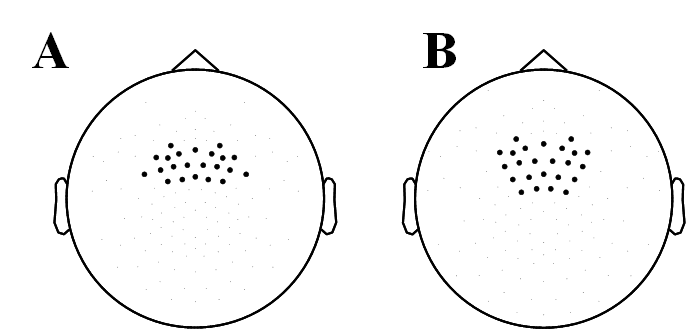
**

**Figure S4. Electrodes selected for additional ERP (A), and TFR (B) statistics analysis.**

**Control study to control the effect of stimulus structure on the omission response**

**Participants**

Ten healthy right-handed volunteers other than those who participated in the main experiment, with inclusion criteria similar to that described in the manuscript, participated in this follow-up study.

**Experiment design**

For this experiment, we used four stimuli: the standard stimulus, the rhythm deviant stimulus, the omission stimulus (omission I), and an additional omission stimulus in which the last chord of the rhythm deviant was silenced (omission II).

The stimuli were delivered in the context of an oddball paradigm. The experimental session consisted of three blocks. The first included high-probability standard stimuli (p = 85%, 595 trials) interspersed with the omission I deviant (p = 15%, 105 trials). The second block included high-probability rhythm deviant stimuli as standard stimuli (p = 85%, 595 trials) interspersed with the omission II deviant (p = 85%, 595 trials). The third block was the same as the first, except that the rhythm deviant was used as the deviant stimulus. Omission I was presented after repetitive presentation of the standard condition, whereas Omission II was presented after repetitive presentation of the rhythm deviant condition. In the main experiment (as well as one of the blocks in the control study), the frequent stimulus was the standard condition, consisting of three chords of 600ms, 300ms, and 300ms. The first and second chords arrived at the same time in different conditions and what changed was the arrival time (or absence) of the last chord. Therefore, the rhythmic tree and structure of omission II are the same as that of omission I. However, the time where the subjects expected the arrival of the last chord is different between omission I and omission II, according to the frequent stimulus (auditory examples are added as SI material).

The order of the deviant stimuli in each block was pseudo-randomized among the standard trials, enforcing three to seven standard stimuli between successive deviant trials. These blocks were presented randomly. Stimuli were delivered through two speakers at 65 dB SPL using Psychtoolbox MATLAB. Participants sat in a comfortable chair in dim light and were instructed to watch a silent movie (March of the Penguins, Warner, ASIN B000BI5KV0). The total duration of the experiment was ~42 min.

**EEG acquisition and preprocessing and ERP analysis**

All the EEG acquisition and preprocessing steps were the same as those described in the main manuscript. In addition, the same procedure was applied to create the ERP results and perform the statistical analyses.

**A**


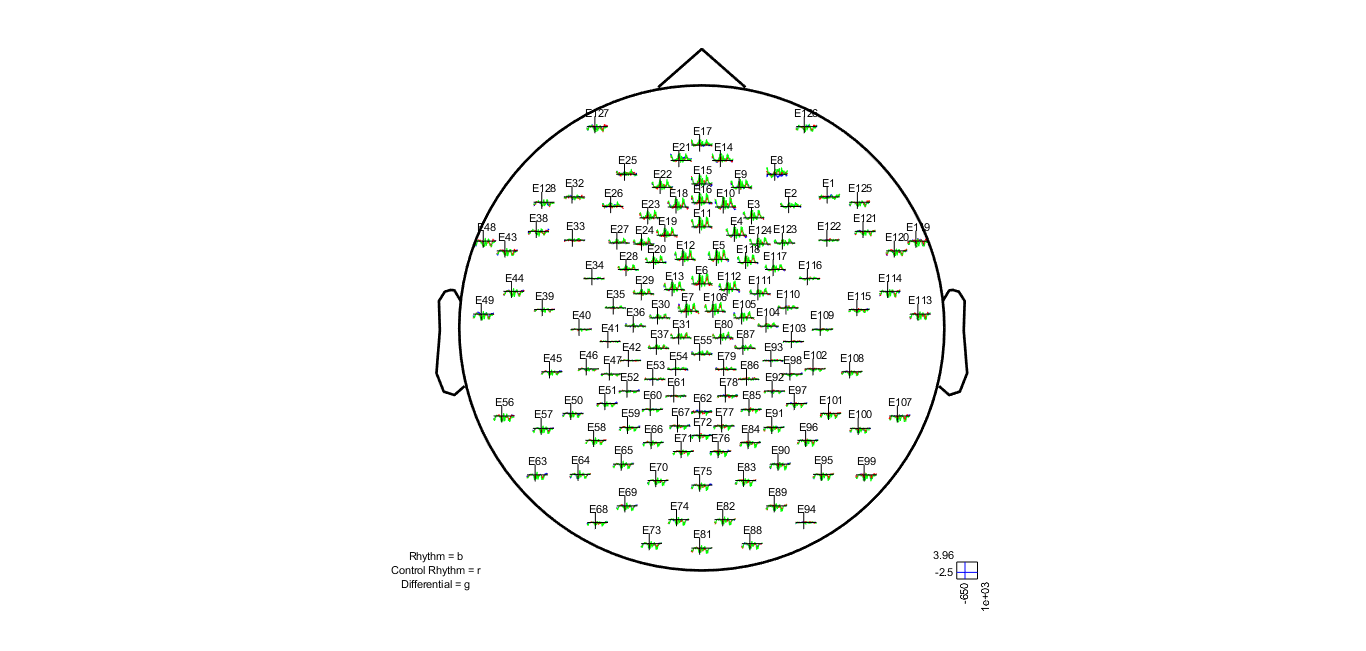


**B**


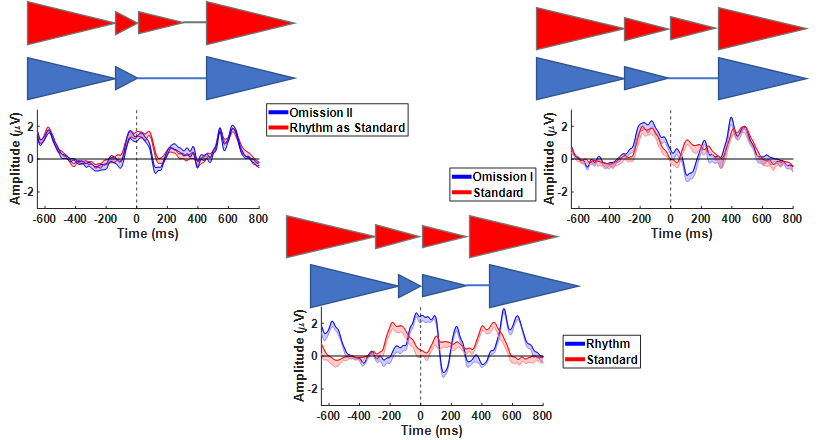


**Figure S5. Event-locked analysis of control blocks. (A) Event-locked analysis of rhythm and omission II conditions. The onset of the deviant chord was set to zero and the next trial started at 450 ms. For all conditions, the baseline was set to 250 to 450 ms from the onset of the first chord. In the follow-up control experiment, the rhythm deviant condition again elicited an MMN response followed by a P3a component. However, the omission II deviant only elicited an MMN response with its timing matching the MMN of rhythm deviant, without a P3a component. (B) Event-locked analysis of each block separately. The onset of the deviant chord was set to zero and the next chord started at 450 ms for rhythm deviant, omission II deviant, and rhythm as standard condition, and at 300 ms for omission I and standard conditions.**

**Phase-amplitude coupling comparing the rhythm deviant condition and shuffled surrogate data**

**
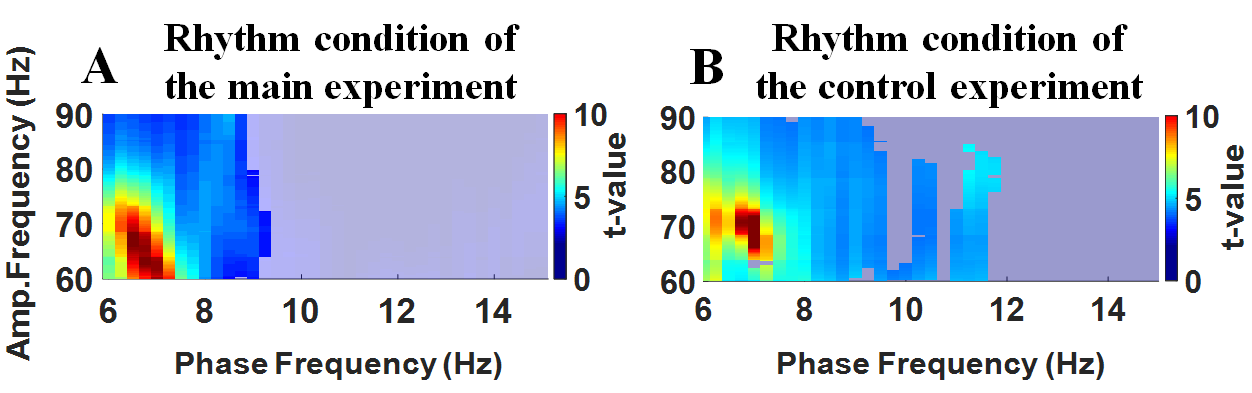
**

**Figure S6. Cluster-based permutation results on phase-amplitude coupling over the 450-ms window, comparing the rhythm deviant condition in the main (*p* = .002, corrected) (A), and control (*p* = .023, corrected) (B) experiment with shuffled surrogate data. The grey regions correspond to frequency pairs for which the permutation analysis did not show significant PAC.**

**Analysis of the pitch deviant condition**

The pitch deviation in this experiment did not elicit a P3a response (see below), as in the case of the omission response, which can be considered a relatively smaller deviation compared to the rhythm deviation. However, since no psychophysical scale was created in this study to evaluate the perceptual distance between conditions, we cannot conclude that the reason for lack of a P3a response is the same as those for the omission condition. Further analysis of the “what” aspect of the stimuli requires manipulations of the chord pitch in different degrees and comparison of the neural response, which was not intended in the context of the current experiment. Considering the experimental design, we cannot conclude that the observed high-frequency oscillatory activity in response to rhythm deviant (and its absence in the pitch deviant condition) is characteristic of the neural response exclusive to rhythm deviations. Pitch direction, the melodic content of the stimulus, and the degree of deviation might also impact the elicited neural response.

***
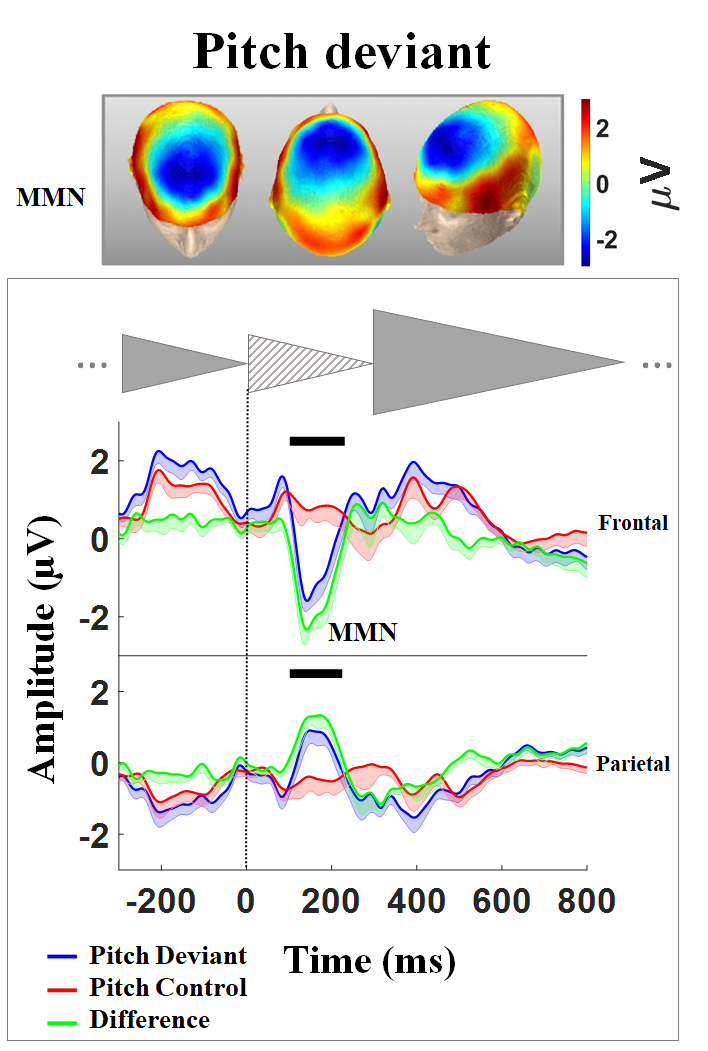
*Figure S7. Grand average of ERP (-SE) for the pitch deviant condition, the control condition, and their difference over frontal and parietal clusters. The onset of the deviant chord was set to zero and the next trial started at 300 ms. For this condition, the baseline was set to 250 to 450 ms from the onset of the first chord. The black bars over the ERP figures represent the time intervals of significant difference between the deviant and control conditions (*p* = 002 for frontal cluster and *p* = .004 for the posterior cluster, marked according to cluster-based permutation analysis). The topography of significant time window is shown in the box.**

***
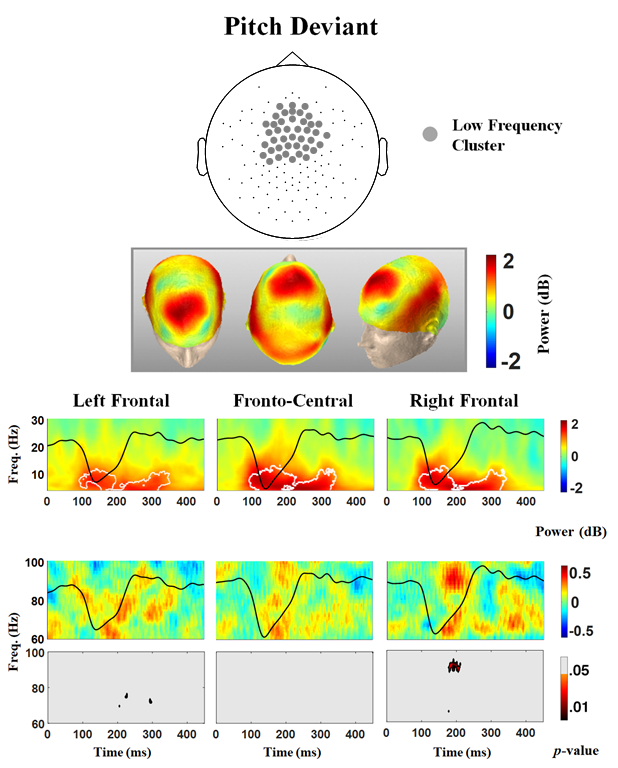
*Figure S8. Event-locked analysis of the pitch condition. The average TFR locked to the beginning of the pitch deviant. The corresponding ERP of the ROI is superimposed on each TFR to better illustrate the results. A white contour indicates the statistically significant changes from the control condition. Low-frequency cluster: 98 to 336 ms, *p* = .001, corrected. The figures below the high-frequency TFRs show the uncorrected p values corresponding to the comparison between the deviant and control conditions (paired-sample t-test). The electrodes' topographical distributions belonging to the significant low-frequency cluster are specified on the head map on top. The topographical distribution of the average power over the frequency and time window corresponding to each cluster is presented in the box.**
